# Screening of exon 11 of BRCA1 gene using the high resolution melting approach for diagnosis in Moroccan breast cancer patients

**DOI:** 10.1186/s12885-015-1040-4

**Published:** 2015-02-25

**Authors:** Meryam El Khachibi, Brehima Diakite, Khalil Hamzi, Abdallah Badou, Mohamed Amine Senhaji, Amina Bakhchane, Hassan Jouhadi, Abdelhamid Barakat, Abdellatif Benider, Sellama Nadifi

**Affiliations:** 1 Genetics and Molecular Pathology Laboratory, Medical school of Casablanca, Casablanca, Morocco; 2 Department of Oncology, Ibn Rochd University Hospital, Casablanca, Morocco; 3 Laboratoire de Génétique Moléculaire Humaine, Département de la Recherche Scientifique, Institut Pasteur du Maroc, Casablanca, Morocco

**Keywords:** Breast cancer, *BRCA*1, Exon 11, HRM

## Abstract

**Background:**

Identification of specific mutations in cancer patients may lead to the discovery of genes, which can affect susceptibility and/or prognosis. It has previously been reported that mutations in *BRCA1* and *BRCA2* genes are linked to breast cancer. Here, we evaluated the use of the High Resolution Melting (HRM) approach to screen for mutations in exon 11 of *BRCA1* gene in Moroccan patients.

**Methods:**

HRM analysis was used to screen exon 11 from 71 breast cancer patients in order to detect different variants. Conventional Sanger sequencing was used to confirm the presence of possible mutations. Distribution of different SNPs was determined by SNaPshot analysis software.

**Results:**

In order to assess the efficacy of the HRM approach to screen for mutations, especially in diagnosis, we first used two samples with previously known mutations, “2924delA and 3398delC”. Indeed, these previously known sequence variants were detected by the HRM approach and yielded melting curves with atypical shape relative to wild-type control sequences. We then analyzed, 69 samples from breast cancer patients using the HRM method, and were able to detect two samples with atypical curves. Sequencing of the two samples, using the conventional Sanger approach, confirmed the presence of the same SNP (c.2612C > T) in both samples.

**Conclusions:**

Our results strongly suggest that the HRM approach represents a reliable and highly sensitive method for mutation scanning, especially in diagnosis.

## Background

*BRCA1* is a tumor suppressor gene located on chromosome 17, position 17q21 in humans. It is comprised of 24 exons spread over 81 kb of DNA, 22 of which are coding exons. These exons encode a transcript of about 7000 bp translated into a protein of 1863 amino acids [1]. Exon 11 of *BRCA1* is a large central exon of 3426 bp. This exon represents 60% of the coding sequence.

Nowadays, it is common knowledge that germ-line mutations of the *BRCA1* gene are high risk factors for developing breast cancer [2]. Since the identification of this particular gene two decades ago [3], it is now frequently used in prognosis worldwide, in women with breast and ovarian hereditary predisposition syndrome [4].

Although Sanger sequencing remains the most reliable technique to identify sequence variants, this approach is costly and time consuming. In order to establish an accurate prognosis in breast cancer patients through the identification of *BRCA1* sequence variants, a number of specific screening procedures, which are both cost- and time- effective have been developed [1].

In Morocco, conventional sequencing techniques have been used to sequence *BRCA1* [5,6]. However, sequencing is quite expensive and since Morocco is a low-income country, most of its patients can’t afford this type of check-up.

The HRM method is a scanning technique that enables mutation scanning and amplification to be performed readily, and in one step [2]. This is a method in which only primers and fluorescent DNA binding dye are used, and no sample processing (after PCR amplification) is required [7]. It is important to note that the HRM technique also allows heteroduplex detection.

Several studies have been conducted to scan *BRCA1* exons by the HRM approach [2,8-11]. However, to our knowledge, this is the first study performed in Morocco using the HRM approach to screen the *BRCA1* gene.

Our aim was to explore the reliability of the HRM approach for the identification of *BRCA1* mutation carriers in exon 11 among family members of an index patient (IP); and to search for the presence of new genetic variants in the Moroccan population. In the first part of this study, we analyzed and detected, using the HRM approach, variants from samples of patients presenting with breast cancer. In the second part, we corroborated the results (obtained by the HRM method) using the standard Sanger sequencing technique.

## Methods

### Patients and samples

We started our work with two previously sequenced, positive controls (2924delA and 3398delC). 71 patients, found positive for breast cancer through clinical and histological examinations, were recruited from private and public oncology centers of Casablanca from 2009 to 2010. Five more donors were used as negative healthy controls.

Experiments performed in this study were evaluated and approved by the Ethic Committee for Biomedical Research in Casablanca (CERBC) of the Faculty of Medicine and Pharmacy (N° 121). For this purpose, a written consent (including the agreement to publish clinical data) was given by each study participant (patient and control).

DNA was extracted using the phenol-chloroform method as previously described, [12] and quantified using the NanoVue™ Plus Spectrophotometer (GE Healthcare, UK).

### HRM protocol

Assays were performed in 96 well plates. Amplification by PCR, of exon 11 of *BRCA1* was performed using primers reported by P. D. Murphy in 2005 (see Table 1) [13] using a 7500 Fast Real-Time PCR system (AB Applied Biosystems, USA). Analysis of the obtained curves was performed using the 7500 Fast System SDS v2.0.1 software.

**Table 1:** **List of primers used to amplify the** ***BRCA1*** **gene exon 11**

Gene		Sequence	Taille
BRCA1	Forward primer	5’ CCACCTCCAAGGTGTATCA-3’	372 bp
exon 11 A	Reverse primer	5’ TGTTATGTTGGCTCCTTGCT-3’	
BRCA1	Forward primer	5’ CACTAAAGACAGAATGAATCTA-3’	400 bp
exon 11 B	Reverse primer	5’ GAAGAAGCAGAATATTCATCTA-3’	
BRCA1	Forward primer	5’ TGATGGGGAGTCTGAATCAA-3’	400 bp
exon 11 C	Reverse primer	5’ TCTGCTTTCTTGATAAAATCCT-3’	
BRCA1	Forward primer	5’ AGCGTCCCCTCACAAATAAA-3’	400 bp
exon 11 D	Reverse primer	5’ TCAAGCGCATGAATATGCCT-3’	
BRCA1	Forward primer	5’ GTATAAGCAATATGGAACTCGA-3’	388 bp
exon 11 E	Reverse primer	5’ TTAAGTTCACTGGTATTTGAACA-3’	
BRCA1	Forward primer	5’ GACAGCGATACTTTCCCAGA-3’	382 bp
exon 11 F	Reverse primer	5’ TGGAACAACCATGAATTAGTC-3’	
BRCA1	Forward primer	5’ GGAAGTTAGCACTCTAGGGA-3’	423 bp
exon 11 G	Reverse primer	5’ GCAGTGATATTAACTGTCTGTA-3	
BRCA1	Forward primer	5’ TGGGTCCTTAAAGAAACAAAGT-3’	366 bp
exon 11 H	Reverse primer	5’ TCAGGTGACATTGAATCTTCC-3’	
BRCA1	Forward primer	5’ CCACTTTTTCCCATCAAGTCA-3’	377 bp
exon 11 I	Reverse primer	5’ TCAGGATGCTTACAATTACTTC-3’	
BRCA1	Forward primer	5’ CAAAATTGAATGCTATGCTTAGA-3’	377 bp
exon 11 J	Reverse primer	5’ TCGGTAACCCTGAGCCAAAT-3’	
BRCA1	Forward primer	5’ GCAAAAGCGTCCAGAAAGGA-3’	396 bp
exon 11 K	Reverse primer	5’ TATTTGCAGTCAAGTCTTCCAA-3’	
BRCA	Forward primer	5’ GTAATATTGGCAAAGGCATCT-3’	360 bp
exon 11 L	Reverse primer	5’ TAAAATGTGCTCCCCAAAAGCA-3’	

In these experiments, we used MeltDoctor ™ HRM Master Mix kit reagents, which contain HRM SYTO9, a DNA intercalating fluorescent agent (BIOLINE, LONDON, UK). The final volume in the reaction mixture is 20 μl (4.4 μl of water, 10 μl of Master Mix, 0.25 μl of each primer (10 μM) and 20 ng of genomic DNA). The HRM protocol was set as follows:

- One cycle of 95°C for 10 min;
- 40 cycles of 95°C for 10 s, 63°C for 30 s and 72°C for 20 s;
- One cycle of melt curve of 95°C for 10 s, 60°C for 1 min, 95°C for 15 s and 60°C for 15 s. In these experiments, we used the same Tm of 63°C for all the primers.

### DNA sequencing

To validate the HRM method, we reanalyzed all of the samples that yielded atypical curves, using conventional Sanger sequencing. These DNA samples were first amplified in a final volume of 25 μl containing: 5× reaction buffer, 1.5 mM MgCl2, 50 μM primers (same primers used for HRM method), 0.25 U Taq polymerase (BIOLINE, LONDON, UK) and 50 ng of genomic DNA.

All the PCR products were treated with exonuclease I and shrimp alkaline phosphatase enzymes prior to sequencing according to the following protocol: 37°C for 40 min and 80°C for 15 min. Then, the obtained PCR products were sequenced using the forward primer, the BigDye Terminator v 1.1Standard Kit (Applied Biosystems, Foster City, CA, USA), and the sequencher 3100 ABI Applied Biosystems. With regard to the analysis, we used the Applied Biosystems SeqScape Software v2.5 SNaPshot analysis.

## Results

**Figure 1:**
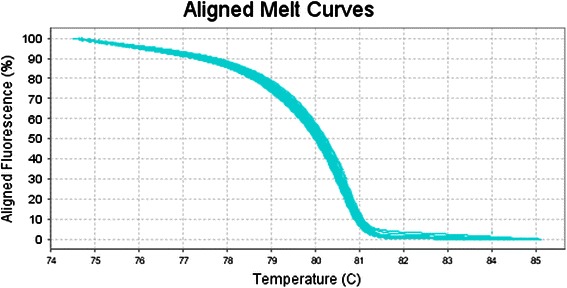
**Aligned melt curves of 71 patients.**

**Figure 2:**
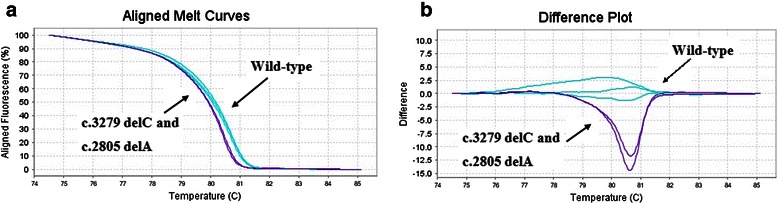
**Difference plots of region H of the** ***BRCA1*** **gene exon 11 between wild-type and c.3279delC and 2805delA mutations (a and b).**

**Figure 3:**
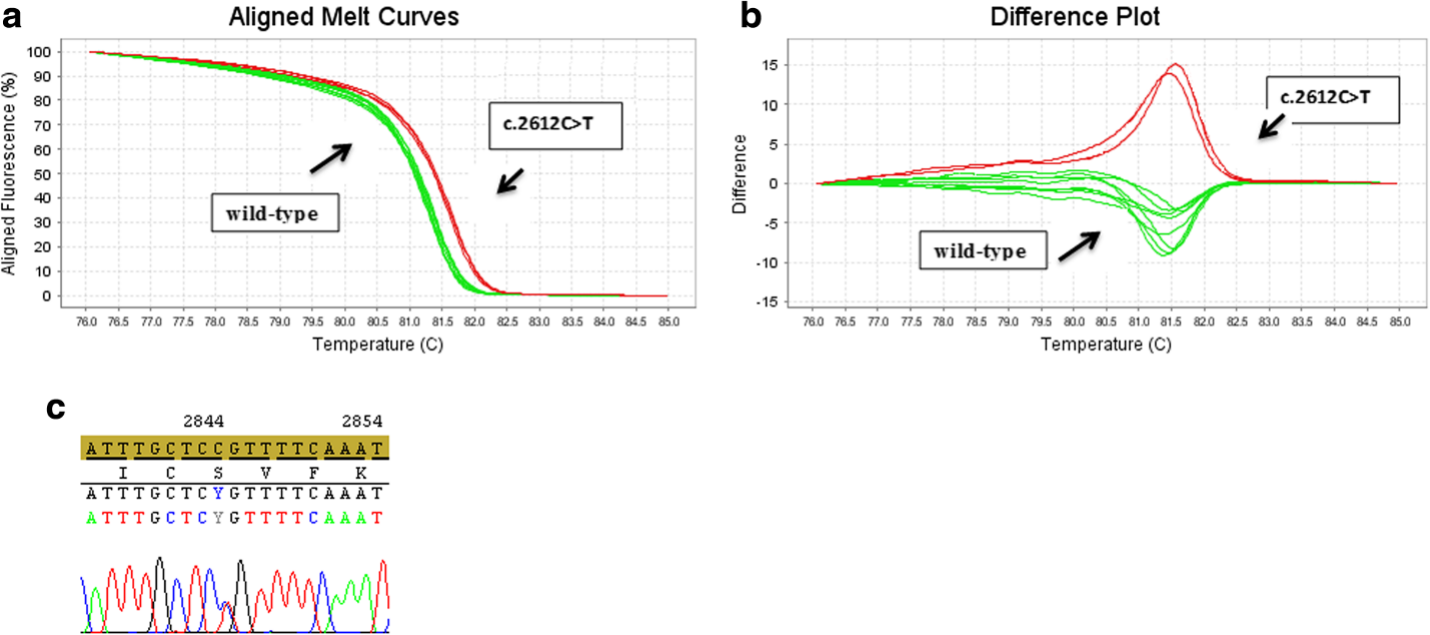
**Difference plots of region G of the** ***BRCA1*** **gene exon 11 between wild-type and c.2612C > T SNP (a; b),** **Electropherograms showing sequencing results for c.2612C > T SNP (c).**

The first step of this work was to validate the HRM approach for the entire sequence of the Exon 11 of *BRCA*1 gene. In order to accomplish this, we used DNA samples from five healthy control donors to amplify all these indicated regions. We also used two positive control DNA samples, in which mutations had previously been detected within the region H of exon 11 (see Table 1) [5]. As expected, all sequence variants in which mutations were previously detected yielded melting curves with abnormal shapes relative to wild-type DNA. In order to corroborate our observation, we sequenced the two samples that exhibited atypical curves, and found that these samples possessed a SNP. Indeed, The B, C, D, E, F, I, K and L regions of exon 11 (see Table 1) did not show atypical curves (Figure 1); whereas the G and H regions of exon 11 (see Table 1) exhibited different atypical curves (Figures 2 and 3). All of these variants have been sequenced except for the samples Br 23 and Br 24, already known to carry mutations in the region H of exon 11 (see Table 2 for details).

**Table 2:** **All mutations and SNPs detected by the HRM approach were validated using Sanger sequencing method**

Gene	Patient code	Exon	HRM result	Sequencing result	Mutation type	Systematic nomenclature	SNP	Manifestation, age at diagnosis	Family history	Ethnic origin
BRCA1	Br 23	11 H	Positive	Positive	c.2805delA	2924delA	_	BC, 41y	M aunt, BC 42 y	Berber
	Br 24	11 H	Positive	Positive	c.3279delC	3398delC	_	BC, 32y	Mother, BC 49 y	Berber
	Br 123	11 G	Positive	Positive	_	_	c.2612C > T	BC, 64y	No Family History	Berber
	Br 12	11G	Positive	Positive	_	_	c.2612C > T	BC, 27y	No Family History	Arab

BC: Breast Cancer.

Our work has shown that the HRM approach is sensitive, specific and straightforward. This method would also help to avoid costly systematic sequencing. In fact, sequencing, in this case, would be performed only for regions with atypical curves.

## Discussion

In order to detect potential mutations and, at the same time, avoid sequencing the entire *BRCA*1 gene, several alternate methods have been suggested to screen this gene. For instance, De Leener, et al. used the Protein Truncation Test (PTT), denaturing Gradient Gel Electrophoresis (DGGE) and denaturing High-Performance Liquid Chromatography (dHLPC), followed by specific region sequencing [8]. While all these methods were considered to be relatively sensitive, these approaches, especially the PTT, were shown to be unable to detect all of the mutations [11]. The purpose of our study was to evaluate the efficacy of the HRM method in screening large sequences for potential mutations.

The High Resolution DNA Melting Analysis was first described by Zhou et al. [14], where the authors analyzed the ΔF508 mutation associated with the Mucoviscidosis disease. These authors also analyzed, using the same approach, other mutations found in the factor V gene: F508C variants in exon 10; and G551D, G542X, and R553X variants in exon 11.

Regarding *BRCA*1 gene screening by the HRM technique, several groups have reported, in agreement with this work, that this method enables the detection of variants [1,2,8,10,11,15,16]. In these reports, positive controls were also used to confirm the specificity and sensitivity of this technique [1,2,8,10,11,15,16]. In 2009, De Juan, et al. used the HRM method followed by sequencing to scan for mutations in the *BRCA*1 and *BRCA*2 genes [11]. Furthermore, in 2011, the same team reported various advantages of the HRM analysis, and confirmed that this method is much faster than Conformation Sensitive Gel Electrophoresis (CSGE) screening [4]. Altogether, these reports are in agreement with this study, and indicate that the HRM approach is suitable as a primary screening method of large DNA sequences, especially in diagnosis.

## Conclusion

In the present study, we show that the HRM approach allows for the screening of mutations across large DNA sequences. DNA samples that produce plots which are distinct from the wild-type, should be sequenced to confirm and identify the specific mutations, or SNPs. Finally, we demonstrate that the HRM approach, which is very sensitive, specific, cost-effective, and fast, can be used efficiently, especially in diagnosis.
